# Magnetization Transfer to Enhance NOE Cross‐Peaks among Labile Protons: Applications to Imino–Imino Sequential Walks in SARS‐CoV‐2‐Derived RNAs

**DOI:** 10.1002/anie.202015948

**Published:** 2021-05-04

**Authors:** Mihajlo Novakovic, Ēriks Kupče, Tali Scherf, Andreas Oxenfarth, Robbin Schnieders, J. Tassilo Grün, Julia Wirmer‐Bartoschek, Christian Richter, Harald Schwalbe, Lucio Frydman

**Affiliations:** ^1^ Department of Chemical and Biological Physics Weizmann Institute of Science 7610001 Rehovot Israel; ^2^ Bruker (UK) Ltd. Banner Lane Coventry UK; ^3^ Institute for Organic Chemistry and Chemical Biology Center for Biomolecular Magnetic Resonance Johann Wolfgang Goethe-University 60438 Frankfurt/Main Germany

**Keywords:** 2D NMR spectroscopy, CEST, NOESY, RNA, SARS-CoV-2

## Abstract

2D NOESY plays a central role in structural NMR spectroscopy. We have recently discussed methods that rely on solvent‐driven exchanges to enhance NOE correlations between exchangeable and non‐exchangeable protons in nucleic acids. Such methods, however, fail when trying to establish connectivities within pools of labile protons. This study introduces an alternative that also enhances NOEs between such labile sites, based on encoding a priori selected peaks by selective saturations. The resulting selective magnetization transfer (SMT) experiment proves particularly useful for enhancing the imino–imino cross‐peaks in RNAs, which is a first step in the NMR resolution of these structures. The origins of these enhancements are discussed, and their potential is demonstrated on RNA fragments derived from the genome of SARS‐CoV‐2, recorded with better sensitivity and an order of magnitude faster than conventional 2D counterparts.

## Graphical abstract

An approach to enhance the sensitivity of 2D NOESY correlations among labile protons is introduced and exemplified with experiments on RNA fragments derived from the SARS‐CoV‐2 genome and with other samples. The approach leads to experiments that are ca. 10‐fold faster and 3‐fold more sensitive than conventional counterparts. The mechanism of this sensitivity boost is explained, and its strengths and limitations are analyzed.

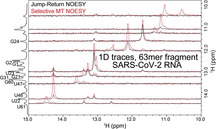

## Introduction

Two‐dimensional homonuclear NMR correlations based on the Nuclear Overhauser Effect, so‐called 2D NOESY experiments, are uniquely endowed to elucidate the structures of biomolecules under near‐native, physiological conditions.[^1^, ^2^, ^3^, ^4^] These experiments rely on detecting magnetization transfers occurring upon taking the spins (typically ^1^Hs) off‐equilibrium, and letting them cross‐relax within a dipolar‐coupled spin network over the course of a mixing time.^[5]^ Despite being routinely performed, 2D NOESY suffers from notoriously low signal‐to‐noise ratio (SNR), requiring hours or even days of signal averaging to detect the cross‐peaks containing its valuable information. Detecting off‐diagonal NOESY peaks between distant sites in a macromolecule ‐which is a crucial requirement to obtain high‐resolution structures‐ becomes even more challenging when probing labile protons that, in addition to cross‐relaxation with their neighbors, undergo chemical exchange with the surrounding solvent. Imino protons in nucleic acids are typical examples of such systems, yet similar cases arise with hydroxyl groups in saccharides, RNAs and protein sidechains, as well as with certain amino and amide groups in proteins. When these are protected from the surrounding aqueous solvent ‐for instance by well‐folded, hydrogen‐bonded structures‐ their resonances are sharp and intramolecular NOESY cross‐peaks detectable. However, when residing in unfolded or exposed regions, peak broadenings can lead to very weak or undetectable cross‐peaks. Looped PROjective SpectroscopY (L‐PROSY)^[6]^ is a recently‐introduced approach to 2D NOESY/TOCSY that attempts to alleviate these problems by turning chemical exchange into a source of repolarization. Thus, instead of using a single mixing period for implementing the homonuclear transfers, L‐PROSY repeats multiple shorter mixing periods ‐all of them carrying a fresh portion of magnetization from the labile protons encoded by spin evolution, thanks to re‐exchanges with the solvent. Cross‐peaks originating from labile→non‐labile transfers can then build‐up with the (faster) initial rate characterizing cross relaxation; this principle was shown to successfully enhance cross‐peaks originating from a variety of labile sites in proteins, sugars and nucleic acids. An even more efficient version of this approach has more recently been proposed based on a Hadamard‐type selective encoding departing from a traditional t_1_ time‐domain incrementation.^[7]^ It was shown that whether involving multiple selective inversions or frequency selective saturation procedures of the labile protons, the ensuing Hadamard‐encoded Magnetization Transfer (HMT) experiment^[8]^ could accelerate conventional NOESY or TOCSY experiments that possessed comparable SNRs by nearly two orders of magnitude.

Despite their remarkable sensitivity gains, both L‐PROSY and HMT demand that the magnetization exchange be established between two distinct spin reservoirs: one is a “donor” pool whose polarization is depleted by radiofrequency (RF) pulses while being replenished by exchange with the solvent; the other is an “acceptor” pool that remains altogether unperturbed, apart from the transfers driven by polarization differences between the two pools. The latter can thus efficiently “accumulate” the transferred information, whose originating chemical shifts are subsequently decoded by either Fourier (vs. t_1_) or Hadamard transformations. This is easily achievable when attempting to establish amide→side chain correlations in proteins, hydroxyl→aliphatic correlations in sugars, or ‐in the cases that will be of prime interest here‐ imino→amino/aromatic/ribose protons in nucleic acids. Important problems arise, however, where the correlations are being sought *within* the same pool of protons. Such is the case for instance encountered when mapping imino‐imino correlations in RNAs: NOESY cross‐peaks arising from guanosine and uridine imino protons contain are the fundamental basis of any sequential RNA assignment, as they carry crucial information about nucleobase pairs.[^9^, ^10^, ^11^, ^12^, ^13^] Clearly, neither L‐PROSY nor HMT can help to address this kind of problem: L‐PROSY because it would encode the targeted spins repeatedly, thereby leading to multiple harmonic resonances after FT; Hadamard because its procedure creates constantly changing combinations of saturated/unsaturated spins, whose cross‐relaxation ‐depending in turn on the spins’ polarization differences‐ becomes entangled in a complex way with the Hadamard encoding process itself.

Driven by the substantial gains exhibited by the L‐PROSY and HMT experiments, the present study seeks an alternative that would still provide more efficient correlations *within* pools of labile sites, as compared to a conventional NOESY. We propose a simple solution based on selective magnetization transfer (SMT) experiments, where peaks are saturated one‐by‐one using tailored, band‐selective RF pulses. When the resulting spectra are subtracted from similar acquisitions where the frequency of the selective saturations is placed symmetrically vis‐à‐vis water, cross‐relaxation patterns similar to those extracted from 2D NOESY but with substantial sensitivity gains, could be obtained. These gains were found dependent on the solvent exchange rate with a given site as well as on the saturation parameters used, in a manner that was well reproduced by numerical Bloch‐McConnell‐Solomon equation models.[^14^, ^15^, ^16^, ^17^] The ensuing experiment, which in some ways is reminiscent of CEST,^[18]^ led to two‐fold enhancements when applied to the amide region of ubiquitin, a well‐folded protein, and to ten‐fold enhancements when used to correlate the hydroxyl resonances of myo‐inositol. However, the best evidence of the approach's usefulness was observed when probing the imino‐imino correlations[^9^, ^19^, ^20^] in RNAs. For a model 14mer model system and for two fragments taken from the SARS‐CoV‐2 RNA genome, the experiment provided all the cross‐peaks observed in jump‐return (JR) NOESY,^[21]^ within ca. 1/10^th^ of the acquisition time. More importantly, SMT identified cross‐peaks from and to rapidly exchanging imino sites that were altogether missed in the conventional NOESY. Full details of the experiment's setup and processing (including sequences and processing pipelines) as well as extensions to more complex scenarios, are presented below.

## Results and Discussion

### Theoretical Considerations

As this study derives from the recently described HMT proposal,^[8]^ it is illustrative to analyze the pros and cons of this Hadamard encoding, when applied to multiple resonances whose mutual NOE correlations are being sought. In the HMT experiment ca. half of the protons are simultaneously perturbed, with each proton being addressed in ca. half of the Hadamard increments. The choice of which ^1^Hs to address is done according to the demands of the Hadamard matrix, which takes into account the need to achieve linear independency between the weightings imparted to the various peaks, while assuming that these weightings will not affect the intensities of the non‐addressed protons. The latter premise, however, will be broken when setting up a Hadamard reconstruction among mutually interacting sites. This can be appreciated from a simple two‐site, two‐scans saturation experiment. The associated Hadamard encoding matrix can be written as $H_{2}\;=\left(\begin{array}{cc} 1 & 1\\ 1 & 0 \end{array}\right)$ , where rows represent the scans, columns represent the two sites A and B being addressed, 1 means that a specific site is saturated, and 0 that it is not saturated. The detected signal will be proportional to the sites’ magnetization, plus cross‐relaxation contributions that will depend on the degree of the polarization imbalance within the spin pair. The signal for the two scans can thus be written as:
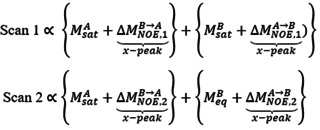



where each bracket denotes the contributions of sites A and B, and the cross‐peak information is marked. Note that since the saturation of the peaks is uneven in the two experiments, their NOE‐derived cross‐peak intensities will not be the same. Hence, its Hadamard‐based reconstruction will yield a mixture of saturation‐weighted NOE contributions, which varies from scan‐to‐scan. In the case of multiple spin pairs this mixture becomes complex, and cross‐peak contributions hard to analyze even under idealized (full saturation) conditions. Supporting Information Figure S1A illustrates this by showing the dependence of these transferred magnetizations between two cross‐relaxing labile ^1^Hs (assumed for simplicity to be imino/amide protons) with respect to a number of relevant parameters ‐saturation time, saturation field and solvent exchange rate. As can be seen from these solution of a Bloch‐McConnell‐Solomon equations model involving three‐spin system (one $H_{A}^{N}$ , one $H_{B}^{N}$ and a 2000‐fold times more abundant water population $H^{w}$ ), the buildup of the cross‐peak extracted from a Hadamard data transform will in general be not monotonic vs. all these parameters. By contrast, if throughout the experiment one of the labile protons is always kept unperturbed ‐i.e., if in the jargon above an $H_{-2}\;=\left(\begin{array}{cc} 0 & 0\\ 1 & 0 \end{array}\right)$ scheme is used‐ an efficient magnetization transfer can be achieved. This is illustrated by the simulations in Supporting Figure S1B, which depict the results of what then effectively becomes a saturation transfer difference experiment.^[22]^

It is enlightening to examine how the efficiency of this selective magnetization transfer (SMT) experiment will be influenced by the solvent chemical exchange and the cross‐relaxation rates of the donor and acceptor spins. Supporting Figure S2 presents this, and plots it in comparison to the intensities expected under identical conditions in conventional NOESY experiments with different mixing times. The selective MT experiments always show equal or superior cross‐relaxation peaks, with the largest gains achieved arising—as in the HMT experiment—when magnetization is transferred from a fast exchanging to a slow exchanging proton. In such cases an ≈15‐fold signal enhancement is achieved vs. conventional NOESY; however, for the opposite, slow→fast exchanging case, the signals of the SMT and NOESY experiments are essentially equal. This asymmetry is unlike what is observed in conventional 2D NOESY, where cross‐peaks arising from a given pair are similar and depend mostly on the sum of effective relaxation rates for both protons. Moreover, while in SMT experiments cross‐peaks extracted by differences will in general be asymmetric (Figure S2A–S2E), in a majority of cases and for sufficiently long saturation times, these signals will always be larger than in conventional NOESY transfers. A possible drawback of relying on such long MT times might result from an enhanced spin‐diffusion among the protons. However, as shown in Supporting Figure S3, introducing a third proton as a potential spin‐diffusion sink in this Bloch‐McConnell‐Solomon equation model, leads to effects that are ca. an order of magnitude smaller than direct NOE transfers. It also leads to a distinct buildup dependence, which if observable in actual experiments could allow one to distinguish long‐distance connectivities from spin‐diffusion effects. Exact distance quantifications, however, would also require independent knowledge of the exchange rates of the cross‐relaxing sites ‐measurements which can be carried out, but would demand independent experiments.^[23]^

### SMT vs. NOESY vs. HMT—Basic Tests

**Figure 1:**
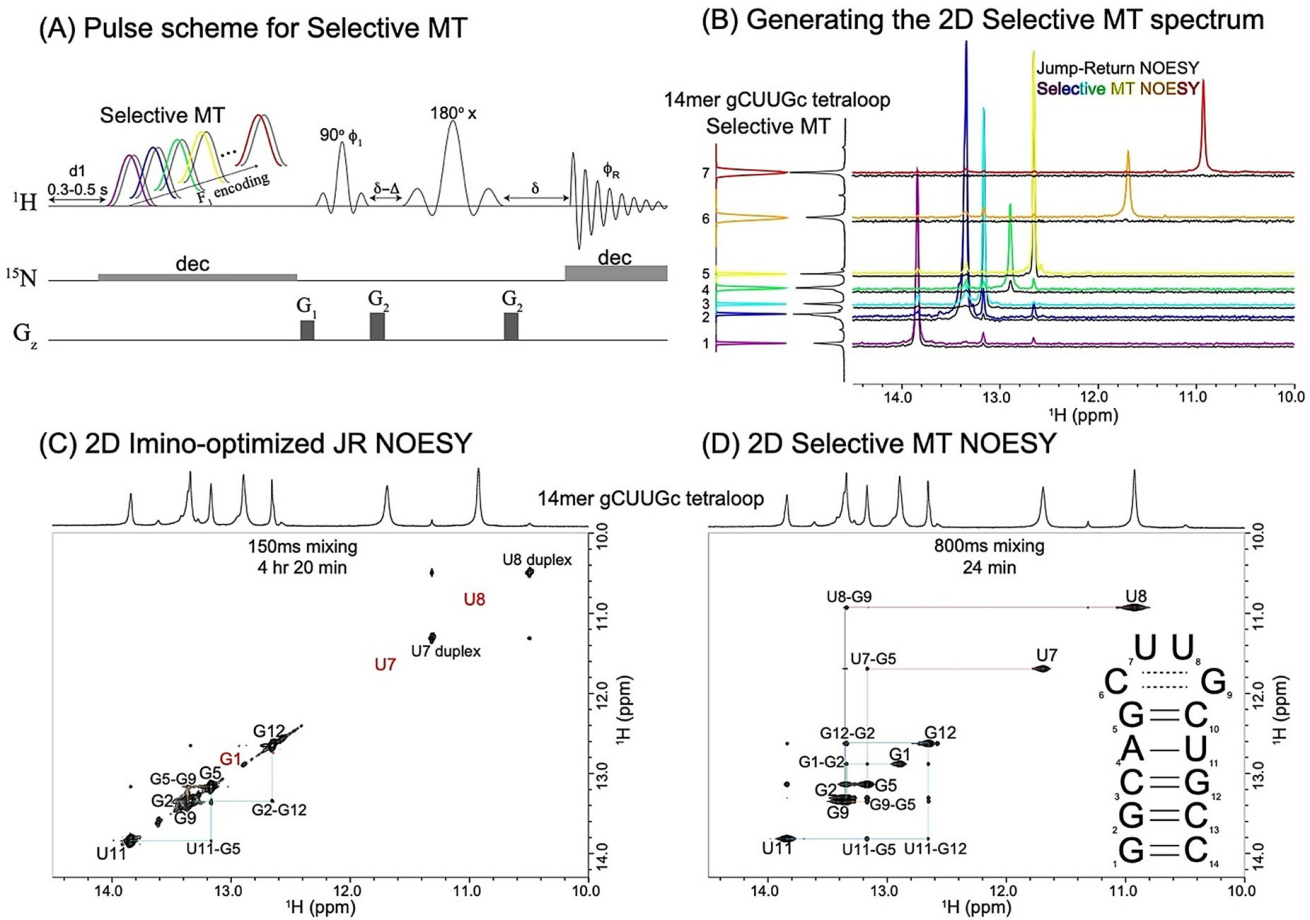
A) Selective magnetization transfer (SMT) pulse sequence: colored shapes correspond to selective on‐resonance saturations of pre‐specified imino resonances; shapes in grey illustrate accompanying reference scans placed off‐resonance and performed consecutively in a phase cycled (add/subtract) manner. Saturation fields can also be tailored for each resonance, based on the width and frequency separation of each peak. Like in HMT, both the encoding and the MT are achieved by the long saturation itself. Since only labile (imino) protons are targeted and detected, a selective spin‐echo (typically involving PC5 pulses for excitation, REBURP pulses for refocusing, and two‐step phase cycle $\phi_{1}=\phi_{R}=(x,-x$ )) was used for the final detection; the delay Δ compensates for the evolution during shaped excitation pulse, while *δ* accounts for the G_2_ gradient duration. As for reasons unrelated to the present study our samples were ^15^N‐labeled, heteronuclear decoupling was applied during the long saturation pulse; to accommodate this while avoiding sample heating, soft on‐resonance ^15^N decoupling was used with specific decoupling offsets chosen according to ^15^N–^1^H correlation spectrum (not shown). ^15^N decoupling was also used during the acquisition, this is all being unnecessary in natural abundance experiments. B) Building up a 2D SMT correlation spectrum by Fourier transformation of the responses of multiple SMT experiments, and their distribution within a 2D plot according to the F_1_‐selected frequencies. To appreciate the enhancements achieved with SMT, horizontal 1D traces extracted from the *≈*11‐fold longer conventional JR NOESY experiment are also shown in black. C) Conventional 2D ^1^H–^1^H NOESY experiment acquired for 14mer gCUUGc tetraloop RNA in 4 hours and 20 min (1D traces shown in B, black). Highlighted in red are sites that give weak or no cross‐peaks in this 2D NOESY, but give cross‐peaks in the SMT. D) Selective MT spectrum acquired on the same sample in 24 minutes (1D traces shown in B, color). Notice that while labile imino hydrogens in G1 (from the beginning of the stem) and U7 and U8 (internal loop) were not detected in the conventional NOESY spectrum due to fast exchange, they provide rather strong correlations in the SMT spectrum. For the fast‐exchanging U7 and U8 sites larger *γ*B_1_ fields were used: 30 Hz, compared to the 8 Hz saturation fields used for the other imino signals. The conventional NOESY used an interscan delay of 1 s; 0.5 s was sufficient for the SMT. As in all remaining 1D projections shown in this paper, the vertical 1D trace in (B) and the horizontal 1D traces in (C, D) correspond to 1D ^1^H results collected using JR water suppression, showing all peaks regardless of their solvent exchange rates. See the Supporting Information (Materials and Methods and Table 1) for additional details on the experimental parameters.

Based on these considerations, the Selective MT pulse sequence shown in Figure 1 A was implemented. In this experiment (see Materials and Methods in the SI for further details), multiple monochromatic frequency‐selective saturation pulses (colored shapes) were generated based on an a priori known 1D spectrum.^[24]^ Their offsets are generated by “clicking” on the chemical shifts of the peaks to be targeted, while B_1_ saturation fields that are specific for each frequency are chosen based on peak broadness and separation from other peaks in the spectrum. This is done to accommodate the predictions from Figure S2 (Supporting Information), whereby peaks that are exchanging more rapidly with solvent ‐a feature usually recognizable by a 1D peak's line broadening‐ can enhance their cross‐relaxation if targeted by stronger saturating field. These monochromatic pulses are executed serially throughout the experiment; given the potential role played by the water in repolarizing the labile spins, each of this scan is followed by an “off” reference acquisition where a saturation pulse of the same B_1_ intensity is applied symmetrically but upfield from the water chemical shift, to compensate for potential water saturation. Such reference pulses (grey shapes in Figure 1 A) were applied in combination with a phase‐cycled receiver phase, so that every (odd) scan involving the saturation of the labile proton of interest, was immediately followed by an (even) acquisition where the reference spectrum is subtracted. To reduce the interscan delays and further increase sensitivity, a selective spin‐echo focusing on the labile protons’ spectral region was utilized for collecting the array of final 1D signals, thereby exploiting the contribution of the solvent exchange to the rapid repolarization of the labile protons.^[25]^ Given the (usually ^15^N‐bound) nature of labile protons the possibility of adding heteronuclear decoupling was also included as an option. All these provisions help increase the signal strength and at the same time reduce potential instrument instabilities and other sources of noise, thus enabling SMT to target the small cross‐relaxation peaks arising along its “indirect domain” ‐despite not enjoying the full multiplexing benefits associated with Hadamard or Fourier processing. Additional information on the nature of the experiments are given in the “Materials and Methods” section of the Supporting Information; further details, including a description of the NMR set‐up, and the pulse sequences needed for SMT's TOCSY and NOESY implementations, can be downloaded from the Bruker User Library at https://www.bruker.com/en/resources/library/application‐notes‐mr/sensitivity‐enhanced‐tocsy‐noesy‐biomolecular‐nmr.html; sequences and parameters are also available for download at https://www.weizmann.ac.il/chemphys/Frydman group/software.

Figures 1 B–D depict how an improved 2D NOESY‐like spectrum results from this procedure, for a model 14mer hairpin gCUUGc tetraloop RNA at 10 °C. This experiment targeted the resonances from the RNA's imino hydrogens, which are key components in arriving at the secondary structure of these molecules. Indeed, the guanosines and uridines in RNA contain single, distinct imino resonances that are in general prone to solvent exchanges in the 10–100 Hz range. These imino peaks will be better protected from exchanges with the solvent when hydrogen bonded in stable base‐pairs; their resonances are then usually sharp, and their NOESY correlations will yield a “base‐pair walk” that is a first step in determining an RNA secondary structure (Figure 1 C). Also valuable are the signals from the imino ^1^Hs residing in stem loops and internal RNA bulges, as these dynamic sites are important motifs in controlling an RNA's folding and provide recognition sites for RNA‐binding proteins.^[26]^ Their signals, however, are usually significantly broadened by exposures to solvent, rarely providing correlations when targeted by ^1^H NOESY experiments. Such absence of cross‐peaks between these imino protons in consecutive nucleobases will break the sequential walk in 2D NOESY assignments, leaving ambiguities that can only be unraveled by undertaking week‐long NOESY acquisitions, or resorting to doubly (^13^C/^15^N) labeled samples. Figures 1 C and D compare 2D NOESY data sets acquired for the aforementioned 14mer using conventional, imino‐optimized, jump‐return 2D NOESY, vs. an SMT spectrum. The conventional 2D NOESY reveals clear nearest‐neighbor correlations for most stable imino groups in the RNA stem, but completely omits the signals from sites undergoing faster chemical exchanges such as G1, U7 and U8. By contrast the SMT spectrum ‐acquired in less than 1/10^th^ of the time it took to collect the conventional 2D NOESY‐ reveals all possible correlations within the 14mer. A clear indication of the superiority of this quicker, more sensitive approach is provided in Figure 1 B, which compares the 1D traces extracted from conventional and SMT NOESY spectra. Notice that while the conventional trace does not provide sufficient correlations for completing a sequential structural walk, the cross‐peaks stemming from the SMT experiment yield an unambiguous assignment of all imino peaks and the confirmation of the RNA's total secondary structure prediction ‐without demanding more involved higher‐dimensional experiments and/or multiple‐labeling schemes. A remarkable example of this is afforded by residues U7 and U8, which while too rapidly exchanging to show up even as diagonal peaks in the conventional 2D JR NOESY, show up and even display minor but observable cross‐peaks to the duplex form, in the 2D SMT.

Another demonstration of SMT's superior efficiency vs. conventional NOESY is shown in Supporting Figure S4, which presents a similar comparison for the same 14mer but recorded at 25 °C—where even faster chemical exchanges between the imino protons and water rob the conventional NOESY from most of its information; on the other hand, these faster exchanges barely affect SMT's correlation performance.

It is enlightening to compare the experimentally obtained cross‐peak patterns in this model RNA, upon using the HMT and the selective SMT encoding schemes. As mentioned in the previous paragraph, the varying initial conditions with which scans are repeated in the full Hadamard encoding can lead to confusing cross‐correlations, including null or even negative cross‐peaks in positions where positive peaks are expected. Supporting Figure S5 exemplifies this experimentally for the RNA 14mer: notice that while both HMT and SMT provide more intense peaks than the conventional NOESY in shorter acquisition times, the intensity and even sign of peaks in the HMT trace lacks the consistency required for an imino‐imino assignment analysis. This is particularly true for the faster‐exchanging sites (e.g. U7, U8), where saturation conditions are complex and incomplete. (It is important to point out that this does not invalidate the use originally meant for the HMT experiment,^[8]^ which was establishing NOESY/TOCSY correlations between inequivalent labile/ non‐labile proton pools.)

Before concluding this 14mer analysis it should be noted that correlations arise in this 14mer's SMT spectrum that are not in line with the canonical structure of the hairpin. These involved cross‐peaks between G1 and the imino sites in G12 and in G5, which arose at all fields (14.1 T, 23.5 T), temperatures (2–25 °C) and $\gamma B_{1}$ fields (10–100 Hz) assayed, and showed normal build‐ups indicative of a genuine NOE between the sites. Presumably, we ascribe this to a feature of the sample preparation conditions, leading to the coexistence of the canonical hairpin with other minority forms, where magnetization between G1, G5 and G12 is relayed efficiently. Further research into this is under way.

### SMT vs. NOESY: Assessing SARS‐CoV‐2 RNA Fragments

**Figure 2:**
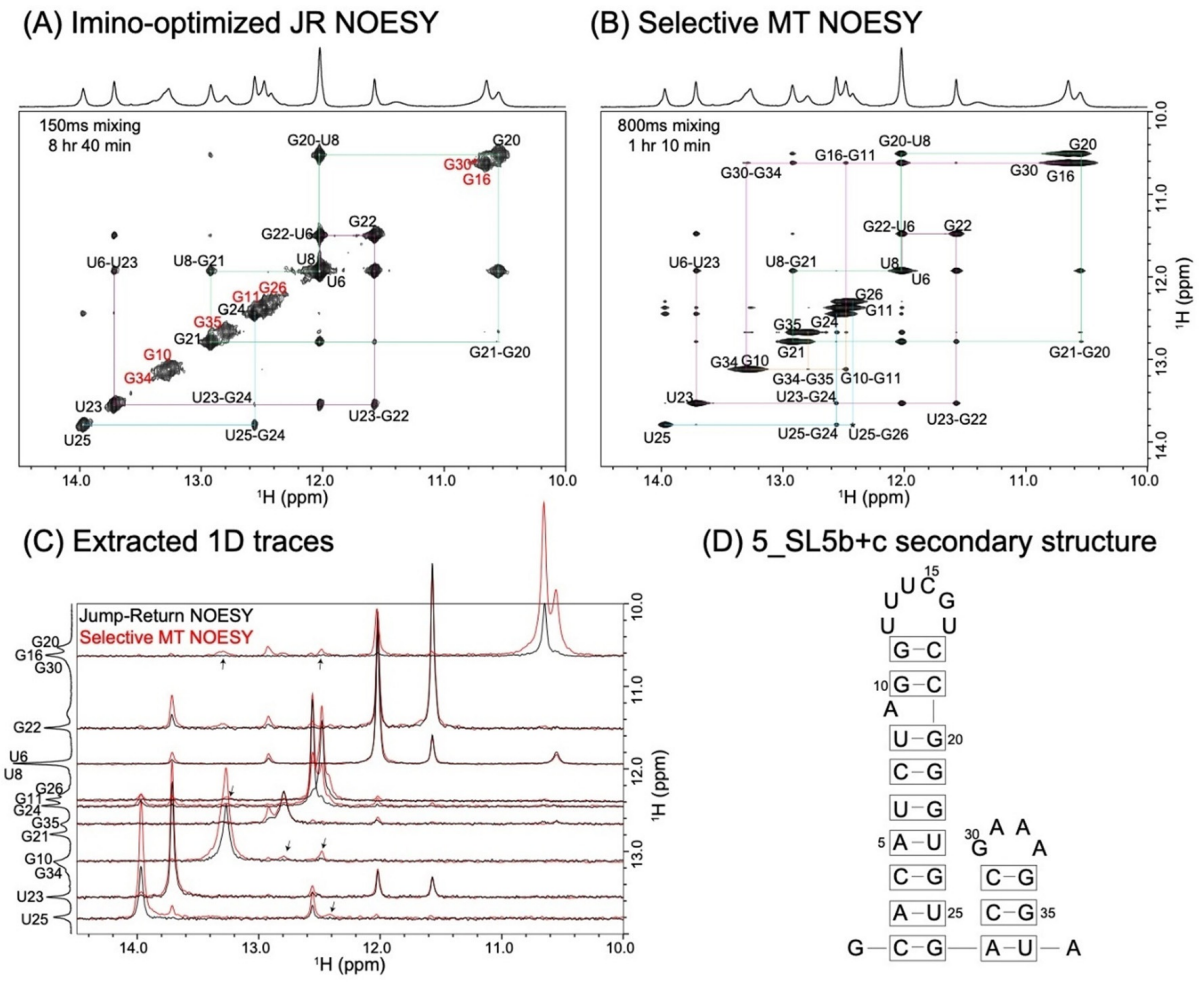
A) Conventional imino‐optimized NOESY spectrum acquired for the 5_SL5b+c SARS‐CoV‐2 RNA fragment in (D), with a 150 ms mixing in 8 hours and 40 minutes. Highlighted in red are sites that gave weak or no cross‐peaks in the 2D NOESY experiment, but give cross‐peaks in the SMT. B) Selective MT spectrum acquired on the same sample using an 800 ms saturation time in one hour and 20 minutes. Both spectra were recorded at 1 GHz, and at 2 °C to slow down exchange. Assignments of various imino protons together with their sequential walk, are shown in the spectra. C) 1D traces extracted from the spectra in (A) and (B) at multiple F_1_ chemical shifts, illustrating the enhancements achieved by SMT for selected bases. Cross‐peaks indicated by arrows facilitated assignments of G10, G11, G16, G30, G34 and G35, which was not possible using the conventional NOESY. D) Predicted secondary structure of 5_SL5b+c arising from the SMT experiments, and from the ancillary data shown in Figure S6 (see Supporting Information).

SMT's ability to deliver fast, sensitive imino‐imino homonuclear correlations that are free from the artifacts associated to HMT's Hadamard processing, was exploited to examine two constructs deriving from the 5′‐ end of the RNA genome of SARS‐CoV‐2. This was done within the framework of the covid19‐nmr project (http://covid19‐nmr.de), whose aim is to characterize all of this virus's regulatory RNAs by NMR spectroscopy. Figure 2 compares conventional and SMT 2D NOESY results for 5_SL5b+c, a 37‐nucleotide fragment deriving from the virus. Although secondary structure calculations predict that this fragment possesses well‐defined base‐paired regions (Figure 2 D), its imino protons were found to undergo fast solvent exchange processes (≈100 s^−1^ at 10 °C). In order to slow these down, all data were recorded at 2 °C. Even at this low temperature the conventional NOESY experiment only provided insight about imino protons positioned in the better‐structured portions of the main stem: G10, G11, G34 and G35 nucleobases forming part of more mobile GC base pairs, as well as iminos positioned at/close to the two internal loops, remained unconnected by the conventional imino‐imino correlations. By contrast, SMT delivered a pseudo‐2D spectrum with multiple cross‐peaks for all the sites, providing further insight into connectivity among all imino protons. Figure 2 C illustrates this with cross‐sections extracted for selected frequencies. Although SMT experiments can also provide correlations between the imino sites and other amino, hydroxyl and aromatic protons, we focused the SMT excitation/detection solely on the imino region; this enabled us to avoid potential complications arising from partial excitation of water, ensuring an uncompromised imino ^1^Hs’ repolarization. Instead, HMT was employed to extract correlations between the iminos and amino/aromatic protons. Together with ancillary HSQC and HNN COSY experiments,[^27^, ^28^] HMT provided a plethora of information (Supporting Figure S6A): these acquisitions enabled the assignment of most ^1^H resonances, eliminating ambiguities about the assignment of guanine and uracil resonances in various GC/UA/UG base pairs, and leading to the secondary structure shown on the right of Figure 2.

**Figure 3:**
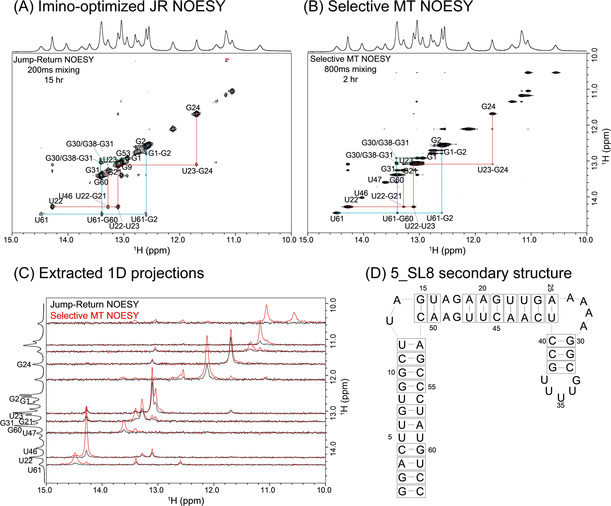
A) Conventional imino‐optimized NOESY spectrum of the 5_SL8 RNA fragment shown in (D), acquired with a 200 ms mixing time in 15 hours. B) Selective MT spectrum acquired using 800 ms mixing in 2 hours. Both spectra were recorded at 1 GHz and at 10 °C. C) 1D projections extracted from the spectra in (A) and (B), comparing traces at the frequencies of multiple residues labeled on the left. D) One of the predicted secondary structures for this 5_SL8 fragment. The slight “off‐diagonal” appearance of some diagonal peaks in panel B reflects minor overlaps from nearby affected resonances.

A more challenging application to reveal a full imino sequential walk is shown in Figure 3, which presents preliminary results on another fragment from the SARS‐CoV‐2 genome. The 5‐SL8 fragment in question has a total length of 63 nucleotides, and numerical models based on the “mfold” ^[29]^ and “kinefold” ^[30]^ RNA secondary structure predictors, suggest multiple different structures and foldings as possible for this sequence—all lying within a relatively small, ≈1 kcal mol^−1^ free energy range. 2D NOESY results are crucial for eliminating these ambiguities; still, a trace recorded at 1 GHz for over 15 hours, gives few correlations for this apparently very dynamic form (Figure 3 A). By contrast, SMT NOESY traces recorded in just 2 hours reveal the presence of several new cross‐peaks (Figure 3 B). Some of these enable a cross‐peak walk leading to unambiguous resonance assignments. Due to the complexity of this structure and due to its putative dynamics between different conformers, however, assignment of all imino protons remains speculative at this stage; it is not included in the Figure, and is likely to demand extension of these experiments to higher dimensionalities, and involve heteronuclear labeling. These experiments are ongoing, as are acquisitions that utilize truncated constructs to simplify the number of assignments needed, while improving the sample's relaxation properties.

## Conclusion

Selective MT was introduced here as an extension of Hadamard Magnetization Transfer, to target NOE correlations that involve labile protons. While HMT provides substantial cross‐peaks enhancements when magnetization from these labile protons is transferred to spectrally distinct protons that were unperturbed during encoding—particularly to non‐labile protons—the same approach ceases to be reliable when the correlations are generated between the addressed resonances. This results from Hadamard encoding imposing multiple alternating perturbations, going beyond the original goals of its multi‐frequency multiplexing. In the case of ^1^H‐^1^H cross‐relaxation this creates artifacts, including attenuated or negative cross‐peaks between the encoded resonances. Selective MT provides a simple solution to this problem which, while giving away Hadamard's multiplexing advantage, can still provide substantial sensitivity gains relative to the standard NOESY experiment. These gains materialize when exchanges with the solvent port fresh polarization onto the targeted site, whose perturbation away from equilibrium by a saturation pulse, translates via dipole‐dipole relaxation into enhanced cross‐peaks with neighboring sites. The rates of solvent exchange and the relaxation properties of the protons on the receiving end of these transfers will limit the potential gains: both numerical simulations and experiments, reveal that the SMT process is most effective when transferring magnetization from fast‐exchanging to slow‐exchanging protons. These sensitivity gains can then magnify NOESY's conventional cross‐peaks by an order‐of‐magnitude, making invisible correlations visible. This was found particularly useful for establishing connectivities among protons in RNAs, where imino sites are known to exchange with water, and where establishing imino‐imino correlations is a key step in structural elucidations. The Supporting Information also illustrates that this can also be the case for NMR studies of polypeptides.

The SMT experiment is easy to set up, and one of its advantages rests in the possibility of using a saturation field $\gamma B_{1}$ set according to the optimal needs of each individual site. Thus, unlike conventional NOESY experiments where a single “one size fits all” mixing time needs to be chosen, SMT allows one to adapt this parameter for each individual site. For the imino sites targeted in the SARS‐CoV‐2 fragments, the broadness of the peaks observed in the 1D ^1^H spectrum gives a good indication of the apparent relaxation, and therefore of the appropriate saturation field that should be applied. For the examples illustrated in this work, these fields ranged from *γ*B_1_/2π=6 Hz for “sharp” peaks, up to ≈35 Hz for the more severely exchange broadened peaks ‐amounting to ca. 20–60 Hz effective spectral resolutions. Given this choice and sufficient spectral dispersion, a simple interleaved acquisition endowed the SMT traces with the information needed to generate a 2D NOESY‐like spectrum.

### Limitations

Despite these stated advantages, the SMT experiment also faces limitations. Like its HMT counterpart it requires the saturation bands to be sufficiently separated; otherwise, peak crowding may confound its results. Hence also like HMT, this experiment will work best when performed at high magnetic fields where site resolution is maximized. This can be appreciated in Supporting Figure S7, where the use of a 1 GHz spectrometer proved crucial for the realization of this kind of experiments on the amide region of a well‐folded 40‐residue polypeptide. The execution of these examinations on a lower, 600 MHz spectrometer called for the use of weaker γB_1_ fields to preserve peak selectivity (data not shown), leading in turn to decreased efficiencies in the magnetization transfers. The potential saturation of multiple overlapping peaks ended up being less of a problem in the RNAs—even for the crowded 5‐SL5 and 5‐SL8 samples. The reason for this lies in the heterogeneity that imino sites in these molecules display in their solvent exchange rates: cross‐talking between peaks that are broad and overlap is then readily revealed by recording the same F_1_ slice with multiple saturation fields, and following the effects on the resulting cross‐peaks intensities. For the above‐mentioned samples the γB_1_ saturation field thus ends up serving, to some extent, as a site separation variable that is not available in conventional NOESY. Supporting Information Figure S8 presents an example of such case, where the parallel behavior of the “diagonal‐peak” saturation and of the concomitant “cross‐peaks” enhancements, enabled the separation of otherwise ambiguous information. As SMT experiments address multiple labile sites there is also the possibility that water‐relayed transfers will confound the cross‐relaxation transfer; yet in our observations, water‐relayed transfers always amounted to ≤0.1 % of the diagonal intensities, even for very fast exchange rates. Similarly, simulations reveal that the effects of relayed transfer via spin‐diffusion will be an order of magnitude smaller than the direct cross‐relaxation transfer. Supporting Figure S3 shows how this could be distinguished from long‐range NOEs according to their distinctive build‐up behaviors. Additional features that could conceivably be incorporated into the proposed Scheme include TROSY^[31]^ optimizations as well as the inclusion of an additional dimension encoding heteronuclear chemical shifts for both HSQC‐NOESY and HNN‐COSY implementations; these and other avenues are currently being explored.

## Conflict of interest

The authors declare no conflict of interest.

## Supporting information

As a service to our authors and readers, this journal provides supporting information supplied by the authors. Such materials are peer reviewed and may be re‐organized for online delivery, but are not copy‐edited or typeset. Technical support issues arising from supporting information (other than missing files) should be addressed to the authors.

Supplementary
