# Supplementary material for: Magnetization Transfer to Enhance NOE Cross‐Peaks among Labile Protons: Applications to Imino–Imino Sequential Walks in SARS‐CoV‐2‐Derived RNAs
Source: Angew Chem Int Ed Engl. 2021 May 4;60(21):11884–91. doi: 10.1002/anie.202015948 (PMC8251384; doi:10.1002/anie.202015948)
Supplement: Supplementary file 1 — Supplementary [file ANIE-60-11884-s001.pdf]

Angewandte Chemie
Eine Zeitschrift der Gesellschaft Deutscher Chemiker

Supporting Information

Magnetization Transfer to Enhance NOE Cross-Peaks among Labile Protons: Applications to Imino–Imino Sequential Walks in SARS-CoV-2-Derived RNAs

Mihajlo Novakovic, Ēriks Kupče, Tali Scherf, Andreas Oxenfarth, Robbin Schnieders, J. Tassilo Grün, Julia Wirmer-Bartoschek, Christian Richter, Harald Schwalbe, and Lucio Frydman**

anie_202015948_sm_miscellaneous_information.pdf

Theoretical aspects of NOESY and of Selective- vs Hadamard-encoded Magnetization Transfers. To assess the SMT process vs conventional NOESY and to describe potential complications of the HMT sequence to correlations between labile protons encoded at the same time, a Bloch-McConnell-Solomon model involving a three-spin system was used. This involved two labile H_A^N, H_B^N protons connected via a generic cross-relaxation process, that were allowed to undergo suitably-population-weighted chemical exchanges with the water proton H^w. The resulting equations can be written as:

$$\begin{aligned}
\frac{dM_y^A}{dt} &= \omega_{1A} M_Z^A - (R_2 + k_A^{sw}) M_y^A + k_A^{ws} M_y^w \\
\frac{dM_z^A}{dt} &= -\omega_{1A} M_y^A - (R_1 + k_A^{sw} + \sigma) M_Z^A + \sigma M_Z^B + k_A^{ws} M_y^w + R_1 M_{eq}^A \\
\frac{dM_y^B}{dt} &= \omega_{1B} M_Z^B - (R_2 + k_B^{sw}) M_y^B + k_B^{ws} M_z^w \\
\frac{dM_z^B}{dt} &= -\omega_{1B} M_y^B - (R_1 + k_B^{sw} + \sigma) M_Z^B + \sigma M_Z^A + k_B^{ws} M_z^w + R_1 M_{eq}^B \\
\frac{dM_z^w}{dt} &= -\left(R_{1_w} + k_A^{ws} + k_B^{ws}\right) M_z^w + k_A^{sw} M_Z^A + k_B^{sw} M_Z^B + R_{1_w} M_{eq}^w
\end{aligned} \qquad \text{(S1)}$$

where M_y^A, M_z^A, M_y^B, M_z^B and M_z^w are the magnetization components of the two labile and of the water spins along specified axis of the Bloch sphere, and M_{eq}^A, M_{eq}^B, M_{eq}^w correspond to the equilibrium magnetizations of these reservoirs (for simplicity normalized to unity). Longitudinal and transverse relaxation rates were calculated as the inverse of the corresponding relaxation times $R_{1/2} = 1/T_{1/2}$, and, in order to account for population differences between the solute and water pools, the exchange rates of the labile and water protons were scaled according to:

$$k^{sw}[solute] = k^{ws}[water] \ . \qquad \text{(S2)}$$

σ in Eq. (S1) represents the dipole-dipole cross-relaxation rates, and can be expressed using spectral densities $\mathcal{J}$ as

$$\sigma = \frac{1}{10} b^2 \left(\mathcal{J}(0) - 6\mathcal{J}(2\omega^0)\right) \qquad \text{(S3)}$$

where $\mathcal{J}(\omega) = \frac{\tau_c}{1+\omega^2\tau_c^2}$ and $b = -\frac{\mu_0}{4\pi}\frac{\hbar\gamma^2}{r^3}$ is the dipole-dipole coupling constant.[1] The strength of saturation fields applied along the *x*-axis were denoted as ω_{1A} and ω_{1B} in Eq. (S1).

The simplest Hadamard matrix H_{-2} needed to distinguish these two sites, solves for the A and B contributions by observing a sum $(A + B)$ and difference $(A - B)$ between saturation effects. It follows from Eq. (1), however, that the magnetizations exchanged between these two labile, cross-relaxing protons in the two Hadamard scan experiments are not the same, leading to multiple unknown NOE terms that cannot be resolved from such system of two equations. In "real world" HMT experiments, where multiple labile proton resonances need to be encoded, the Hadamard approach will thus lead to cross-peaks that become strongly weighted by multiple factors, including the saturation field and duration, and the sites' solvent exchange rates. By contrast, if all the receiving pool is always in thermal equilibrium and a single-site encoding is performed, then "pure" NOE cross-peaks can easily be extracted. To exemplify this, transferred magnetizations between two labile protons were simulated for the HMT and SMT scenarios using

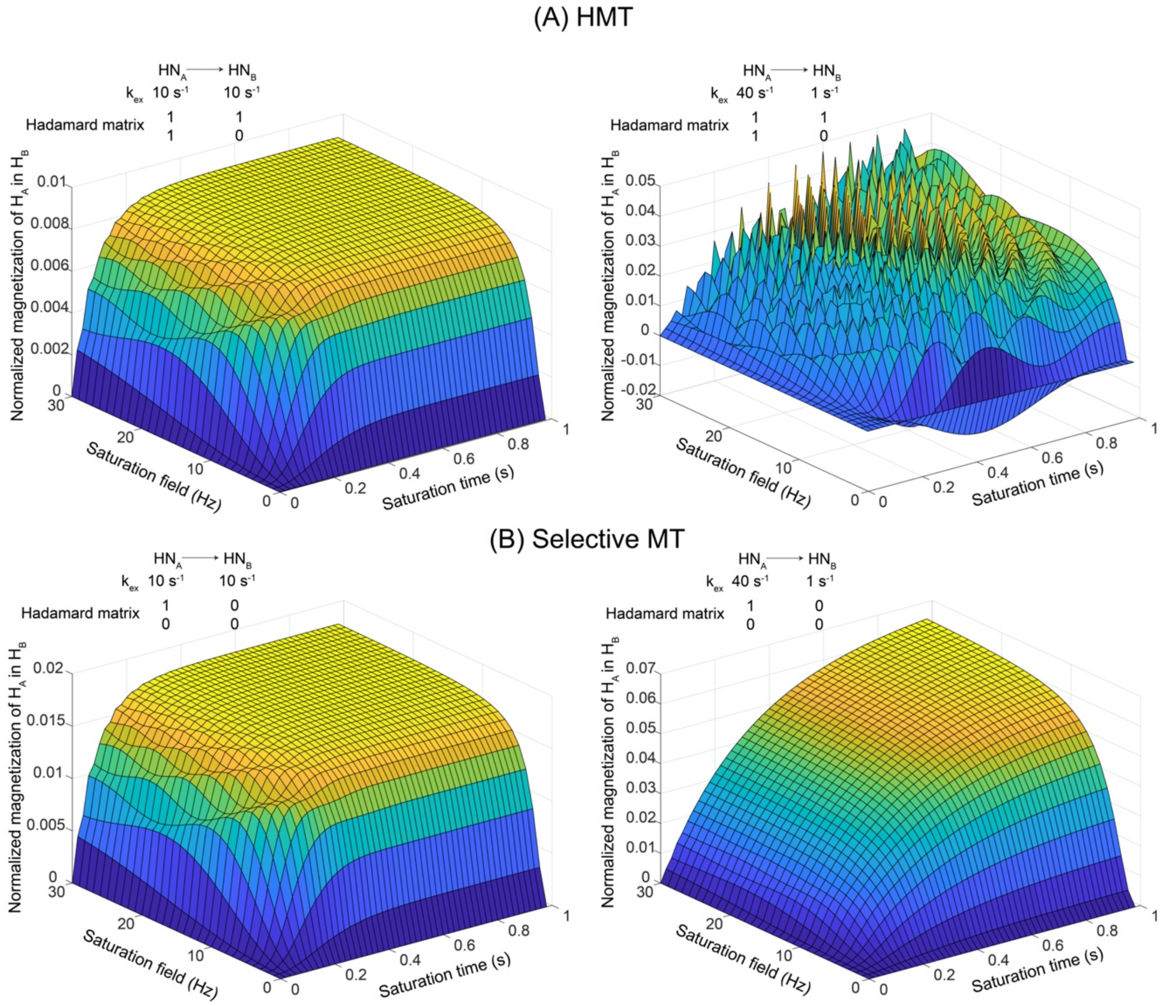

Figure S1. (A) Cross-peak buildup in HMT experiment simulated by Bloch-McConnell-Solomon equations (S1) for two sites H_A^N and H_B^N, possessing different exchange rates with the solvent. Notice the pronounced oscillations present when the two chemical exchange rates are very different. (B) Similar buildups but for a Selective MT experiment. Notice the monotonic buildups with respect to the saturation field and duration, and the different magnetization levels reached for the different exchange cases. Cross-relaxation rates were calculated at 14.1 T for $\tau_c = 5\,ns$ correlation times and $r = 3.3$ Å internuclear distance. Relaxation rates were chosen as $T_2 = 0.3\,s$ and $T_1 = 0.5\,s$; water relaxation constants were taken $T_{2_w} = 0.5\,s$ and $T_{1_w} = 3\,s$. A 2000-fold excess of water was assumed.

the equations above; in all cases, a relaxation-free $\sigma = 0$ situation was used to normalize the results obtained with $\sigma \neq 0$. Figure S1A presents the ensuing cross-peak buildup of spin H_A^N into H_B^N, after encoding and reconstruction using the Hadamard matrix in Eq. (1) of the main text.

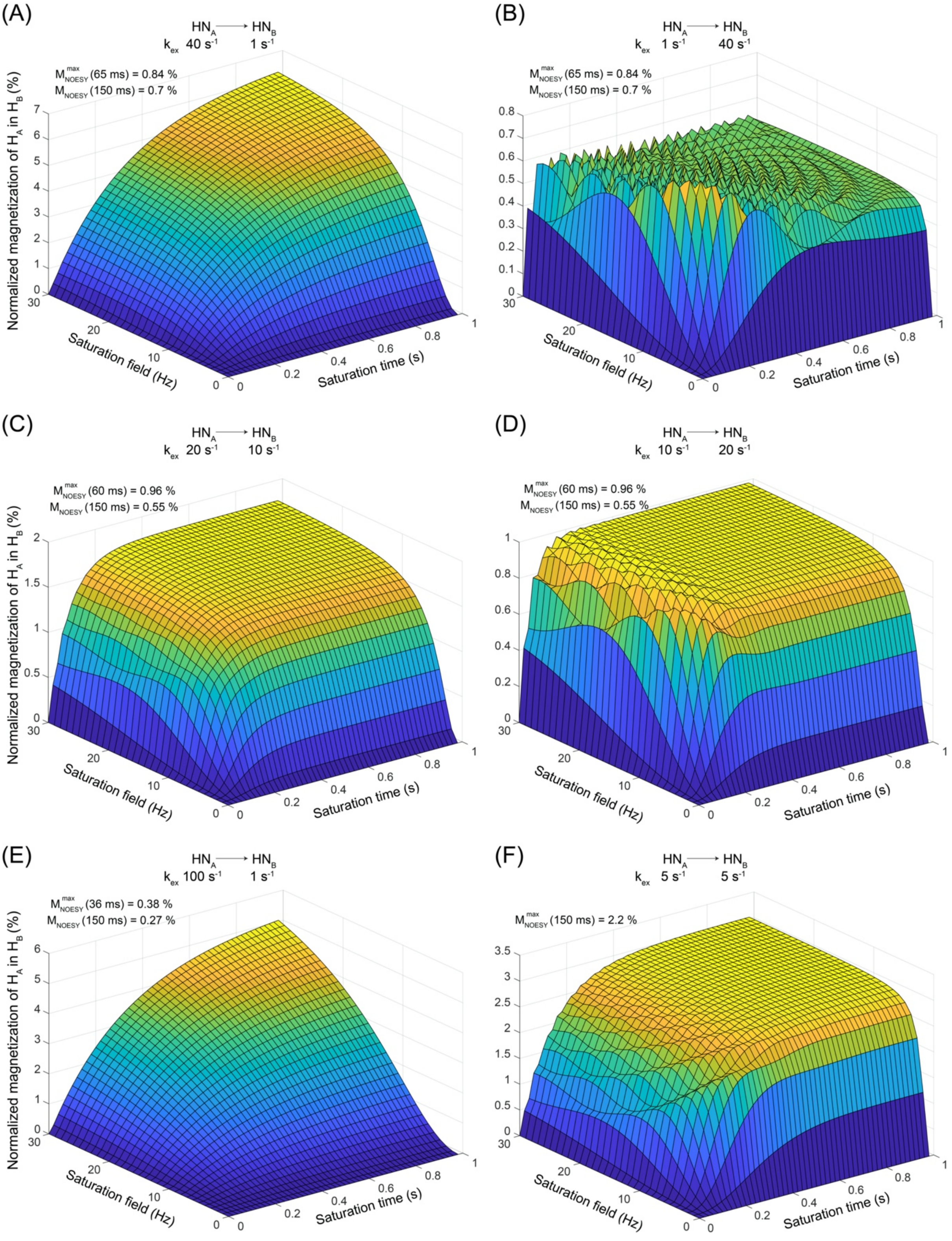

Figure S2. Cross-peak buildups predicted by Eqs (S1) for SMT experiments simulated for the different solvent exchange rates of H_A^N and H_B^N specified in panels (A)-(F). Cross-relaxation rates were calculated at 14.1 T for a $\tau_c = 5\ ns$ correlation time and $r = 3.3$ Å internuclear distance. Relaxation rates were chosen as $T_2 = 0.3\ s$, $T_1 = 0.5\ s$; water relaxation times were set to $T_{2_w} = 0.5\ s$ and $T_{1_w} = 3\ s$. A 2000-fold excess of water was assumed. The insets denote the cross-peak intensities (in % of the diagonal) detected by conventional NOESY for every condition. It follows that Selective MT provides largest enhancements when exchange rate k_A is large and k_B small.

Notice the strong dependence of the isolated "cross-peak" arising from the Hadamard Transform,

when the chemical exchange rates of two spins are different. Actually, when the solvent exchange rates of A and B are the same, then $\Delta M_{NOE,1}^{B\to A}$, $\Delta M_{NOE,1}^{A\to B}$ and $\Delta M_{NOE,2}^{B\to A}$ during the first and second scans of the HMT encoding are close to zero, leaving $M_{NOE,2}^{A\to B}$ as dominant contribution to the NOE effect. This can then be well isolated by the addition/subtraction combination. On the other hand, when the exchange rates with solvent (or when any other relaxation parameter) for the two sites are very different, then a Hadamard transformation yields the complex behavior on the right-hand side of Fig. S1A. As illustrated in Figure S1B SMT solves this complication, yielding predictable, monotonic buildups of the cross-peaks with respect to saturation field intensity and duration and, if performed for sufficiently long, to the highest achievable cross-peak intensities.

Figure S2 further examines this matter, by evaluating the relative SMT efficiencies vis-à-vis conventional NOESY predicted by the above-introduced model, for various combination of exchange rates of the two labile sites. Notice that according to these calculations SMT will not yield symmetric cross-peak information: while it provides efficient transfers to slow exchanging protons regardless of the solvent exchange rate of the donor proton, it is more inefficient the other way around. This reflects the decoherence that fast water exchanges impart on the magnetization on the "receiving end", reducing its memory time and not allowing it to buildup its transferred NOE. Furthermore, notice how the choice of an optimized B_1 saturation field defines the experiment's efficiency: the faster the exchange, the larger the saturation fields needed to saturate efficiently the fast-exchanging protons. By contrast, conventional NOESY cross-peaks are in most cases symmetrical, and a function of the sum of exchange rates of donor and acceptor spin pool.

Finally, to examine potential spin-diffusion effects during the rather long saturation pulse of SMT, Figure S3 shows the effect of adding a third labile proton to the spin system and allowing it to cross-relax with the second –but not to the first– of the protons being correlated. Saturation of H_A^N detected in H_B^N is indeed relayed to H_C^N; nevertheless, the effect of this relayed transfer is rather small compared to the direct NOE: it starts building up much later, and reaches levels that are always an order of magnitude smaller than direct transfer. Given sufficient sensitivity, one could distinguish direct from relayed cross-peaks could by an analysis of the different shapes in their build-up curves.

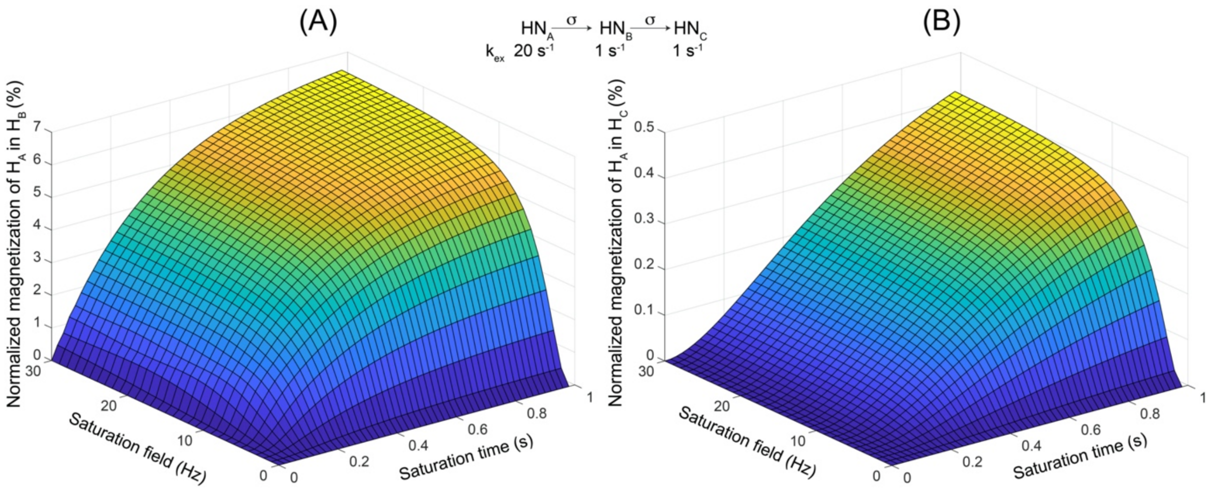

Figure S3. Buildup of magnetization in Selective MT experiments when a third imino proton is included in the model of Eq. S1. (A) Direct MT from H_A^N to H_B^N. (B) Relayed transfer from H_A^N to H_C^N. Other simulation parameters are as specified in Figures S1 and S2.

SMT provides all correlations even when chemical exchange is fast. Complementing Figure 1 from the main text, Figure S4 shows conventional NOESY and SMT acquired on the 14mer hairpin RNA sample at 25 °C, where imino protons are additionally broadened by fast chemical exchange. While the sensitivity of NOESY is greatly compromised compared to its 10 °C counterpart, SMT still provides all the same correlations as at lower temperatures.

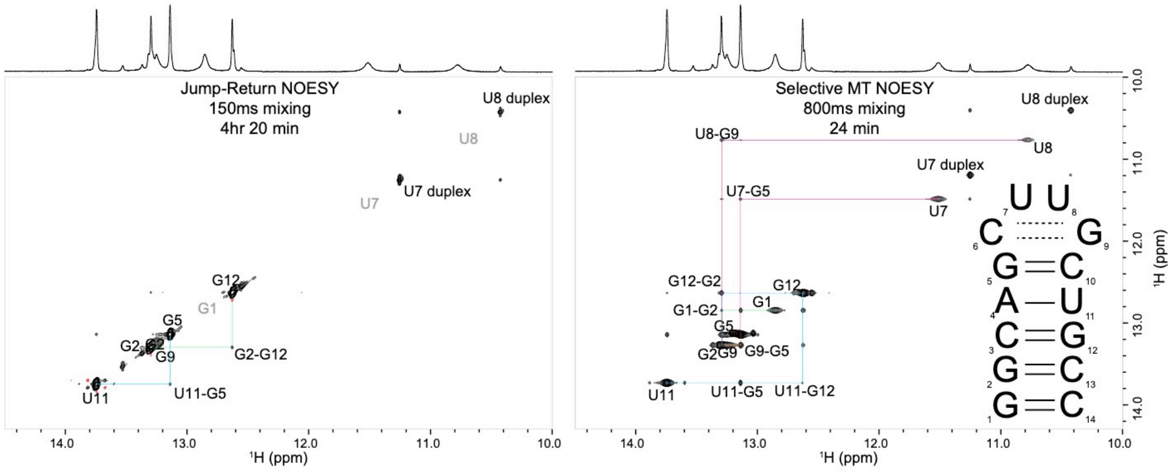

Figure S4. Comparison between conventional and SMT ^{1}H-^{1}H NOESY experiments acquired on the 14mer gCUUGc RNA at 25 °C on a 1 GHz spectrometer. Notice that while G1, U7 and U8 are significantly broadened by solvent chemical exchange and devoid of cross-peaks on the left, SMT still provides all the same correlations as observed at 10 °C.

Experimental comparisons of the cross-peak intensities in NOESY, HMT and SMT NMR. Figure S1 predicted that the cross-peak buildup of peaks in HMT is complex and depends on experimental conditions, relaxation parameters and chemical exchange rates. Figure S5 experimentally illustrates this by presenting extracted slices from imino-imino NOESY correlation spectra acquired using conventional JR NOESY, HMT and Selective MT for the 14mer hairpin sample introduced in Fig. 1, at 10 °C. Conventional JR NOESY yields the scarcest data; although HMT cross-peaks are stronger, they are always less sensitive than the Selective MT's counterparts –in some cases appearing absent or inverted, leading to ambiguous interpretations.

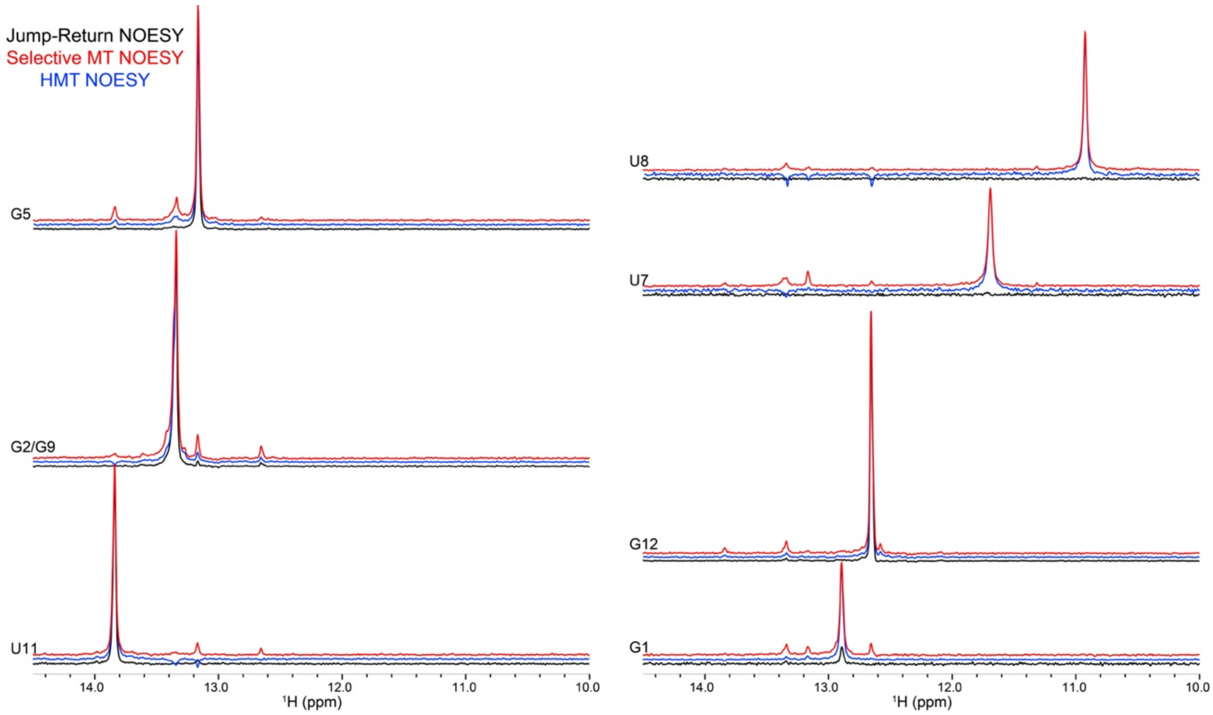

Figure S5. Slices Extracted from 2D NOESY (black), HMT (blue) and SMT (red) experiments for various imino protons in the RNA 14mer of Figure 1. While HMT provides ambiguous correlations and even negative cross peaks between correlated iminos, SMT yields genuine correlations that are always equal or greater than those in the NOESY experiment. SMT data were acquired in 24 minutes; the conventional NOESY took 4 hours and 20 minutes.

HMT imino correlations in the SARS-CoV-2-derived fragments: Corroborating the sequential imino SMT assignment. Supporting Figure S6 shows HMT spectra acquired on 5_SL5b+c and 5_SL8, zooming into spectral regions correlating the imino ^{1}Hs along F_1 with the amino/aromatic protons along F_2 (Figures S6B and S6D). These spectra provided more intense and artifact-free correlations than their conventional NOESY counterparts (Figures S6A and S6C), and were used in the assignments process. Notice that although the HMT spectrum of 5_SL5b+c in Fig. S6 was acquired at 10 ºC, it provided more cross-peaks for fast exchanging imino protons (e.g. G11, G34 and G35), than the conventional NOESY counterpart acquired at 2 ºC –where chemical exchanges are significantly slower. To further aid the assignment of homonuclear ^{1}H-^{1}H correlations, an additional ^{15}N multiple-quantum filter was added in the water-suppression block selectively erasing solely ^{15}N-bound amino protons. Ensuing spectra representing imino-aromatic/ribose protons correlations are shown in red, especially highlighting high enhancements that HMT provided to aromatic protons. This leads to the indicated peak assignment, which corroborate the “NOESY walks” presented in Figures 2 and 3 in the main text. Notice as well that this SNR improvement and additional information is achieved in only ~40 min, compared to acquisitions that are 10-20x longer for NOESY counterparts. Furthermore, since optimized conventional NOESY spectra provide the correlations with amino/aromatic protons by placing these along the F_1 dimension, there are differences in lineshapes and in the cross-peaks close to the water resonance (which were truncated in the HMT due to the Watergate 3919, regions highlighted in yellow) as a result.

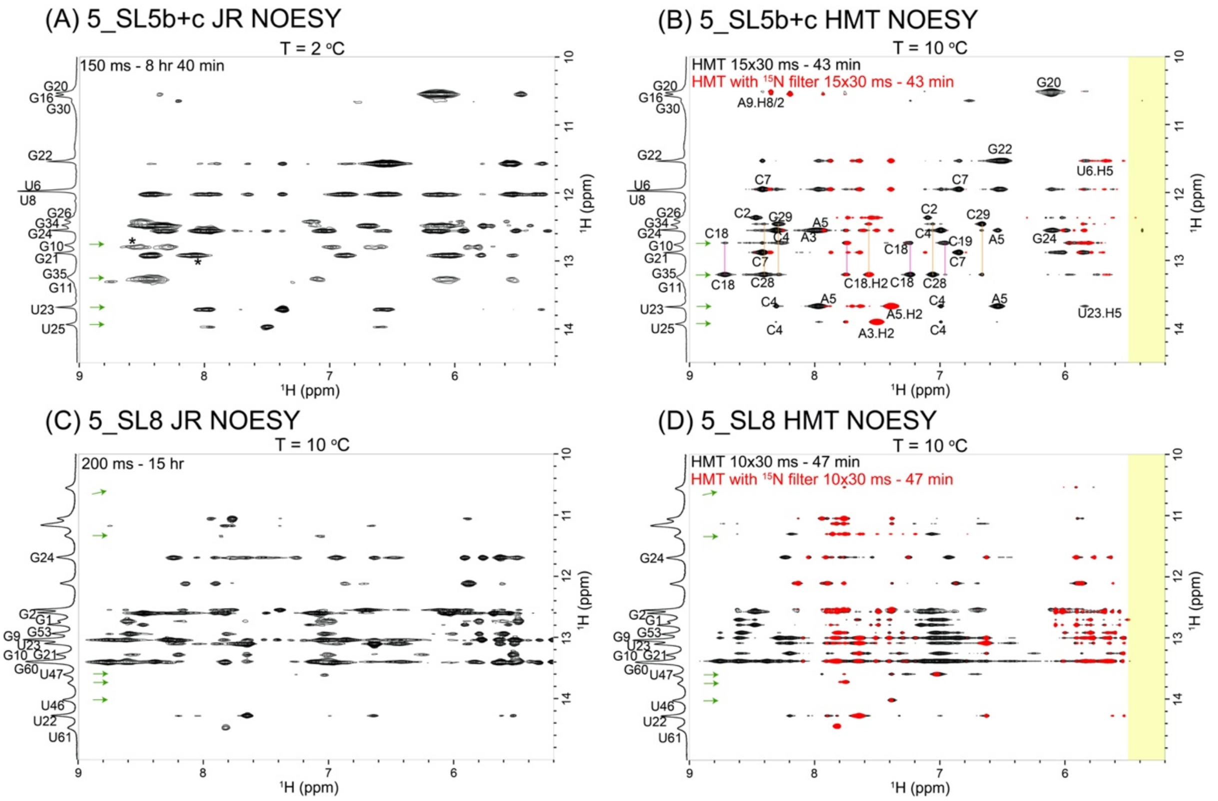

Figure S6. (A) Conventional NOESY experiment acquired for 5_SL5b+c RNA fragment comparing to HMT imino→amino/aromatic/ribose protons correlation in (B) with included assignments and links connecting the common cross-peaks for G10-G11 and G30-G34-G35 that helped the assignment of imino-imino SMT correlations. (C,D) Similar comparison is shown for 5_SL8 RNA fragment. Full assignments of the imino protons are not available for 5_SL8, and therefore are not included in HMT spectrum. Artifacts in the conventional NOESY spectrum (not present in the HMT) are indicated with asterisk. In both HMT panels, spectra shown in red represent an HMT variant with an added ^{15}N multiple-quantum filter that eliminates amino cross-peaks and helps distinguish them from the rest. Pointed with green arrows are imino strips that show few correlations in conventional NOESY while in HMT experiments numerous cross-peaks are detected. Highlighted in yellow are the truncated regions caused by Watergate 3919 water-suppression scheme used in HMT method; this could be significantly improved using other water-suppression schemes (e.g. excitation sculpting) instead. All data were collected at 1 GHz.

SMT in polypeptide elucidations. The examples mentioned in this manuscript's main text focused on RNAs, where the need to target imino-imino NOESY correlations arises as the first step in an NMR analysis. Other instances may arise where, although not so central, correlations within labile sites might still be informative and challenging to obtain. Supporting Figure S7 shows such an example, involving NOESY applications focusing on the 6-10 ppm region of LA5, the ligand binding domain 5 of the low-density lipoprotein receptor LDLR. This is a well-folded protein,[1] and the majority of cross-peaks in this 2D NOESY spectral region arise from correlations between proximate amide sites. Given the well-protected nature of these sites in the protein, their exchanges with the solvent are slow; therefore, little or no sensitivity or acquisition time gains arise from extracting these correlations via SMT. This is clear from an overall inspection of the 2D contour plots in Figure S7, and is further emphasized by a number of representative blue-colored 1D traces shown in this Figure. Interestingly, however, certain strong

off-diagonal peaks arise in the SMT spectrum, revealing correlations that are not visible in the conventional NOE experiments; the red 1D traces in Figure S7 exemplify this. Analysis of these data reveals that the 6 ppm trace shown corresponds to a hydroxyl site, while the other two traces correspond to amides that show up as broader peaks in the protein's HSQC spectrum. It follows also that in instances other than nucleic acids, SMT can provide information unavailable from 2D NOESY NMR –in particular, when entailing exchange-broadened, dynamic protons.

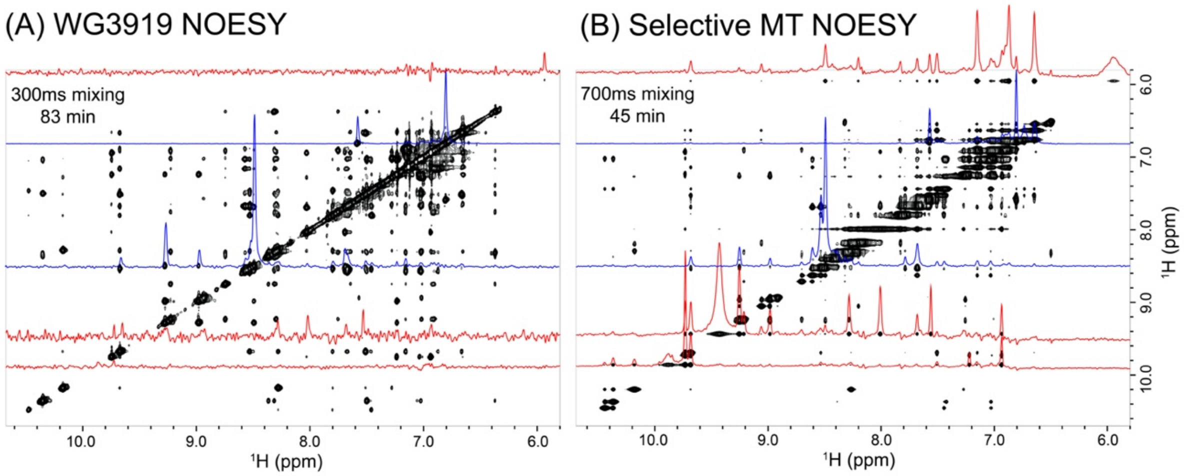

Figure S7. (A) Conventional NOESY spectrum collected using a Watergate 3919 water suppression scheme optimized for the detection of amide protons, acquired on the LA5 protein. (B) SMT NOESY counterpart acquired in ca. 2x shorter acquisition times. Traces in blue illustrate sharp, slowly exchanging protons that were not enhanced by SMT; traces in red are extracted from fast exchanging amides/hydroxyl protons that show multiple correlations in the SMT, but none in the conventional NOESY experiment. All data were recorded at 1 GHz.

On the ability of SMT to use B_1 intensities in order to discriminate cross-peaks originating from overlapping "diagonal" peaks. Spectral resolution can be compromised in SMT by the need to use a sufficiently strong saturating B_1 field to highlight the cross-peaks being sought. In actuality, however, B_1 also provides a variable to "improve" resolution, particularly if cross-peaks arise from overlapping sites possessing differing exchange behaviors. Figure S8A illustrates an example of this for two peaks from the 5_SL5b+c fragment, G16 and G20, that overlap significantly with one other. Saturating each of these peaks will consequently lead to a perturbation of the other peak, creating common cross-peaks that can complicate the individual connectivities. However, because of the sites' different exchange behavior, there is a significant asymmetry of how much irradiation of G16 perturbs G20 and vice versa. One can utilize this to clearly distinguish the origin of cross-peaks resulting from each site, as shown in Figure S8B.

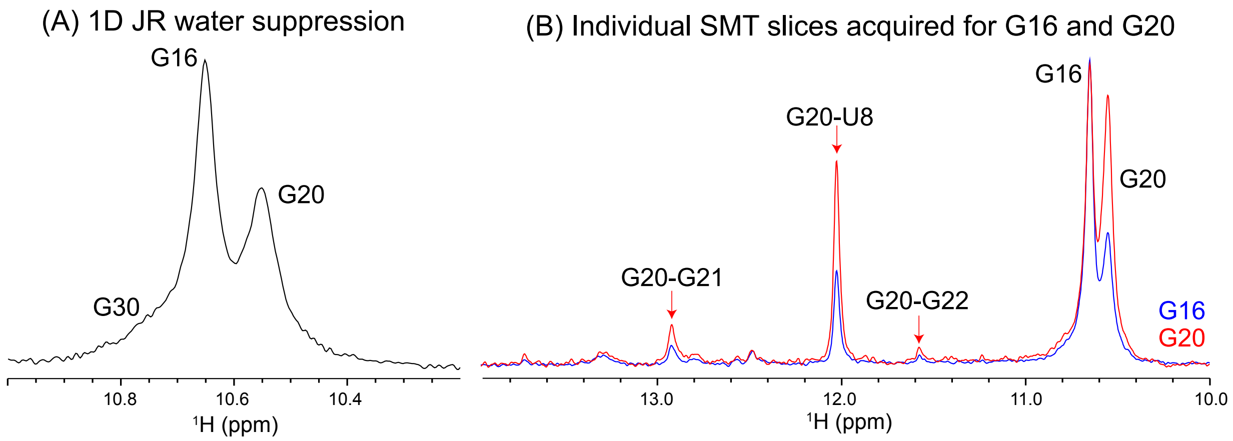

Figure S8. (A) 1D jump-return water-suppressed spectrum of 5_SL5b+c at 2°C illustrating the overlap between iminos G16 and G20. (B) SMT difference experiment slices acquired for G16 (blue) and G20 (red; all data extracted from Figure 2 in the main text) upon using $\gamma B_1/2\pi$=25 Hz, showing how a differential analysis of the changes exhibited by the various peaks can lead to differentiation of cross-peaks and their unambiguous assignment.

Materials and Methods

Sample preparation. The labeled 14mer gCUUGc tetraloop (5'-pppGGCAGCUUGCUGCC-3') was prepared from a linearized plasmid DNA by a run-off *in vitro* transcription using the T7 RNA polymerase.[2] In addition, the plasmid DNA contained a self-cleaving HDV ribozyme to ensure 3' homogeneity. Labeled rNTPs were purchased from Silantes (Munich, Germany). The RNA was also $^{13}C/^{15}N$-labeled (for purposes other than those addressed in this study); it was folded in buffer (10 mM phosphate buffer + 1 mM EDTA pH: 6.4) in 90% H_2O and 10% D_2O by denaturing it for 5 min at 95°C and subsequently slowly cooling down to room temperature. The final concentration of the 14mer in the NMR tube was 1 mM. 5_SL5b+c and 5_SL8 RNAs were produced by T7 polymerase-based *in-vitro* transcription.[3] Template sequences of 5_SL5b+c and 5_SL8 together with the T7 promoter were generated by hybridization of complementary oligonucleotides and introduced into the *EcoR*I and *Nco*I sites of an HDV ribozyme encoding plasmid based on the pSP64 vector (Promega). The recombinant vectors were transformed and amplified in *Escherichia coli* strain DH5α. Plasmid-DNA (8 – 10 mg plasmid per liter SB medium) was purified by Gigaprep (Qiagen) according to the manufacturer's instructions and linearized with *Hind*III (or *Sma*I in case of 3_HVR) prior to *in-vitro* transcription by T7 RNA polymerase (P266L mutant, prepared as described in Guilleres et al.[2] The RNAs were purified as follows: preparative transcription reactions (6 h at 37°C) were terminated by addition of EDTA and RNAs were precipitated with 2-propanol. RNA fragments were separated on 10% (5_SL8) or 12% (5_SL5b+c) denaturing polyacrylamide (PAA) gels and visualized by UV shadowing at 254 nm. Desired RNA fragments were excised from the gel and RNA was eluted by passive diffusion into 0.3 M NaOAc and precipitated with EtOH. Residual PAA was removed by reversed-phase HPLC using a Kromasil RP 18 column and a gradient of 0-40% 0.1 M acetonitrile/triethylammonium acetate. After freeze-drying of RNA-containing fractions and cation exchange by $LiClO_4$ precipitation (2% in acetone), each RNA was folded in water by heating to 80°C followed by rapid cooling on ice. Buffer exchange to NMR buffer (25 mM potassium phosphate buffer, pH 6.2, 50 mM potassium

chloride) was performed using centrifugal concentrators with a suitable molecular weight cut-off membrane. RNA purity was verified by denaturing PAA gel electrophoresis and homogenous folding was monitored by native PAA gel electrophoresis. The final concentration of the SL5b+c and 5_SL8 samples in the NMR tubes were 0.7 and 0.8 mM, respectively.

LA5, the ligand binding domain 5 of the low-density lipoprotein receptor LDLR, was prepared as described by Szekely *et al*[10] at pH 7.4 and concentration 3 mM in 10 mM Tris buffer with 1 mM $CaCl_2$. The protein sample was prepared in H_2O/D_2O (90%:10%) with NaN_3.

NMR experiments. All NMR experiments were conducted on a 1 GHz, 23.5 T Bruker Avance Neo spectrometer equipped with a TCI cryoprobe. Selective MT experiments were acquired using 64-256 scans depending on biomolecule dynamics and sample concentration. The resonances encoded in the SMT were chosen from either 1D Watergate 3919 or 1-1 echo 1D acquisitions, optimized in the RNA cases for detecting signals between 10 and 15 ppm. In most cases 6-10 Hz nutation fields were used for saturation; 20-35 Hz B_1 fields were used to efficiently saturate broader imino resonances. The SMT reference ("no-saturation") scan was set by placing the same B_1 field as adjusted for the "on-resonance" scan, at -5 ppm. The duration of the saturation pulse was set to 0.8 sec, based on average effective polarization recovery times of the labile protons (≈0.1-0.6 sec, dominated by exchange effects). As all samples were also ^{15}N-labeled (again, for purposes unrelated to this work) decoupling was used during saturation this was a low-power garp4 (pw90=500 μs; γB_1=500 Hz)[4] with the list of offsets chosen for every signal as per an available ^{15}N-^{1}H FHSQC[5] spectra (a low resolution 2D spectrum was sufficient for this purpose). NOESY mixing times ranging from 150-200 ms were utilized in the conventional experiments, which included imino-optimized 1-1 echo (jump-return) water suppression;[6,7] for the LA5 sample NOESY with Watergate 3919 water suppression[8,9] and a mixing time of 300 ms were used. All SMT pulses were generated using WaveMaker via the "seq_sl" au-program, that combines a peak list and saturation fields to make the selective RF pulses; spectra were processed using custom-written au-program "proc_seq" directly in Bruker® TopSpin® 4.0.9, apodized with QSINE window functions and zero-filled to 512 F_1 points. Additional experimental details, including a description of the SMT experimental set-up, and pulse sequences for TOCSY and NOESY implementations, can be downloaded for free from the Bruker user library: https://www.bruker.com/en/resources/library/application-notes-mr/sensitivity-enhanced-tocsy-noesy-biomolecular-nmr.html. Sequences and parameters are also available in https://www.weizmann.ac.il/chemphys/Frydman_group/software. Supporting Table S1 summarizes the main parameters used in the various SMT and conventional JR NOESY acquisitions presented in this study.

Supporting Table S1. Parameters used for conventional and SMT NOESY experiments to acquire the spectra presented in the main text.

RNA	14mer		5_SL5b+c		5_SL8b+c	
Parameters	JR NOESY	SMT	JR NOESY	SMT	JR NOESY	SMT
Temperature (K)	283	283	275	275	283	283
SW (ppm)	20.83	20.83	20.83	20.00	20.83	21.73
TD	4096	4096	4096	2560	4096	4096
SW_1 (ppm)	11.50	/	11.50	/	12	/
TD_1	768	8	768	12	512	20
NS	16	128	32	256	64	256
DS	32	32	32	32	32	32
d_1 (s)	1	0.5	1	0.5	1.4	0.5
RG	101	101	101	101	101	101
O_1 (F_2, ppm)	4.693	4.693	4.686	4.686	4.693	4.693
O_1 (F_1, ppm)	8.50	/	8.50	/	9	/
d_8 (NOE, ms)	150	800	150	800	200	800
$\gamma B_1/2\pi$ saturation (Hz)	/	8-30	/	8-25	/	7-25

References

[1] O. Szekely, G. Armony, G. L. Olsen, L. S. Bigman, Y. Levy, D. Fass, L. Frydman, *Biochemistry* **2018**, *57*, 4776–4787.

[2] J. Guilleres, P. J. Lopez, F. Proux, H. Launay, M. Dreyfus, *Proc. Natl. Acad. Sci. U. S. A.* **2005**, *102*, 5958–5963.

[3] J. F. Milligan, O. C. Uhlenbeck, in *Methods Enzymol.*, **1989**, pp. 51–62.

[4] Z. Zhou, R. Kümmerle, X. Qiu, D. Redwine, R. Cong, A. Taha, D. Baugh, B. Winniford, *J. Magn. Reson.* **2007**, *187*, 225–233.

[5] S. Mori, C. Abeygunawardana, M. O. Johnson, P. C. M. Van Zijl, *J. Magn. Reson. Ser. B* **1995**, *108*, 94–98.

[6] V. Sklenar, A. Bax, *J. Magn. Reson.* **1987**, *75*, 378–383.

[7] V. Sklenar, *J. Magn. Reson. Ser. A* **1995**, *114*, 132–135.

[8] G. Lippens, C. Dhalluin, J. M. Wieruszeski, *J. Biomol. NMR* **1995**, *5*, 327–331.

[9] M. Piotto, V. Saudek, V. Sklenář, *J. Biomol. NMR* **1992**, *2*, 661–665

[10] O. Szekely, G. Armony, G. L. Olsen, L. S. Bigman, Y. Levy, D. Fass, L. Frydman, *Biochemistry* **2018**, *57*, 4776–4787.
